# The PI3K-Akt pathway inhibits senescence and promotes self-renewal of human skin-derived precursors *in vitro*

**DOI:** 10.1111/j.1474-9726.2011.00704.x

**Published:** 2011-08

**Authors:** Shuang Liu, Shu Liu, Xinyue Wang, Jiaxi Zhou, Yujing Cao, Fei Wang, Enkui Duan

**Affiliations:** 1 State Key Laboratory of Reproductive Biology, Institute of Zoology, Chinese Academy of SciencesBeijing, China; 2 Graduate University of the Chinese Academy of SciencesBeijing, China; 3 State Key Laboratory of Experimental Hematology, Institute of Hematology & Blood Diseases Hospital, Chinese Academy of Medical Sciences & Peking Union Medical CollegeTianjin, China; 4 Department of Cell and Developmental Biology and Institute for Genomic Biology, University of Illinois at Urbana-ChampaignUrbana, IL, USA

**Keywords:** skin-derived precursors, PI3K-Akt, self-renewal, cellular senescence, adult stem cells, PTEN

## Abstract

Skin-derived precursors (SKPs) are embryonic neural crest- or somite-derived multipotent progenitor cells with properties of dermal stem cells. Although a large number of studies deal with their differentiation ability and potential applications in tissue damage repair, only a few studies have concentrated on the regulation of SKP self-renewal. Here, we found that after separation from their physiological microenvironment, human foreskin-derived SKPs (hSKPs) quickly senesced and lost their self-renewal ability. We observed a sharp decrease in Akt activity during this process, suggesting a possible role of the PI3K-Akt pathway in hSKP maintenance *in vitro*. Blocking this pathway with several inhibitors inhibited hSKP proliferation and sphere formation and increased hSKP senescence. In contrast, activating this pathway with PDGF-AA and a PTEN inhibitor, bpV(pic), promoted proliferation, improved sphere formation, and alleviated senescence of hSKPs, without altering their differentiation potential. Data also implied that this effect was associated with altered actions of FoxO3 and GSK-3β. Our results suggest an important role of the PI3K-Akt pathway in the senescence and self-renewal of hSKPs. These findings also provide a better understanding of the cellular mechanisms underlying hSKP self-renewal and stem cell senescence to allow more efficient expansion of hSKPs for regenerative medical applications.

## Introduction

Among various adult stem cell types within the skin, the newly identified skin-derived precursors (SKPs) have been extensively investigated in recent years because of their multipotent differentiation ability and potential clinical applications. SKPs could differentiate into neurons, glial cells, adipocytes, muscular cell types, insulin-producing cells, and so forth (Qiu *et al.*, 2010; Toma *et al.*, 2001; Guo *et al.*, 2009). Studies also pointed out that murine facial SKPs originated from embryonic neural crest, and dorsal trunk SKPs originated from the somites. SKPs persist into adulthood, with dermal papillae (DP) of hair follicles as one niche for them (Fernandes *et al.*, 2004; Hunt *et al.*, 2008; Jinno et al., 2010). It has recently been shown that SKPs exhibit properties of dermal stem cells that contribute to dermal maintenance, wound healing, and hair follicle morphogenesis (Biernaskie *et al.*, 2009). Therefore, SKPs have great potential in skin and hair follicle reconstitution, and as a donor cell type for the repair of many other tissues. Realizing the therapeutic potential of SKPs in human requires strategies to generate not only a particular functional cell type, but also a considerable cell quantity for transplantation. However, although SKPs have been successfully isolated from human skin (Toma *et al.*, 2005), some fundamental questions remain intangible, especially those concerning their self-renewal regulation. Previously, we found that human foreskin-derived SKPs (hSKPs for short in this article) could not maintain long-term self-renewal in the commonly used suspension culture system, consistent with the report by Gago *et al.* (2009).

In the present study, we explored the regulation of hSKP self-renewal and its underlying mechanisms. We found that hSKPs quickly became senescent under current culture condition, which was at least partially responsible for their loss of self-renewal ability. Senescence and aging of stem cells have been studied, but their regulation remains largely uninvestigated. We focused on phosphatidylinositol 3-kinase (PI3K)-Akt (PI3K-Akt) pathway because of its well-established role in controlling cell proliferation, survival, as well as senescence. For the whole organism, this pathway is strongly linked to aging and lifespan regulation because it can profoundly change the numbers and activity of different types of stem cells (Sahin & DePinho, 2010). In mouse and primate embryonic stem cells (ESCs), activation of Akt signaling is sufficient to maintain their pluripotency (Watanabe *et al.*, 2006). *In-vivo* and *in-vitro* studies revealed a crucial role of the PI3K-Akt pathway in self-renewal and differentiation of neural stem/progenitor cells (Groszer *et al.*, 2001, 2006; Sinor & Lillien, 2004; Gregorian *et al.*, 2009). Because SKPs were first isolated using culture condition for neural stem/progenitor cells and these two stem/progenitor cell types exhibit similar properties, the PI3K-Akt pathway is a good candidate for the regulation of hSKP self-renewal and cellular senescence.

In this study, hSKP senescence *in vitro* was investigated, with a focus on the role of the PI3K-Akt pathway. Our study elucidated the basis for the lack of *in-vitro* expansion of adult hSKPs that were observed by several groups (Joannides *et al.*, 2004; Gago *et al.*, 2009). We also suggested that the PI3K-Akt pathway played a role in the maintaining hSKPs *in vitro* and stimulation of this pathway by PDGF-AA and bpV(pic) provided an improved *in-vitro* expanding condition for hSKPs.

## Results

### Isolation and characterization of hSKPs

The hSKPs can be routinely isolated and cultured from human foreskin. During primary culture, most of dermal cells adhered to culture dishes or died while a portion of cells aggregated to form spheres, which could be passaged by trypsinization and pipetting as shown in Fig. S1A (Supporting information). As reported before, these hSKPs expressed nestin, fibronectin, and vimentin (Fig. S1B). Using reverse transcription–polymerase chain reaction (RT–PCR), we also detected several embryonic neural crest stem cell markers, p75 neurotrophin receptor (p75NTR), Pax3, Slug, and Snail in hSKPs (Fig. S1C), which were also reported to be expressed by hSKPs (Toma *et al.*, 2005).

To test the differentiation potential of these cells, Day 12 hSKPs were differentiated under different conditions for 2–4 weeks. At the end of the assay, cells positive for smooth muscle alpha actin (α-SMA), Oil-Red O, β-III-tubulin, major microtubule-associated protein 2 (MAP2), and S100 protein were observed (Fig. S1D–G), suggesting the differentiation into smooth muscle cells, adipocytes, neurons, and glial cells. The results showed that these hSKPs had multiple differentiation potentials.

### hSKPs quickly became senescent in culture and could not maintain their self-renewal ability

**Fig. 1:**
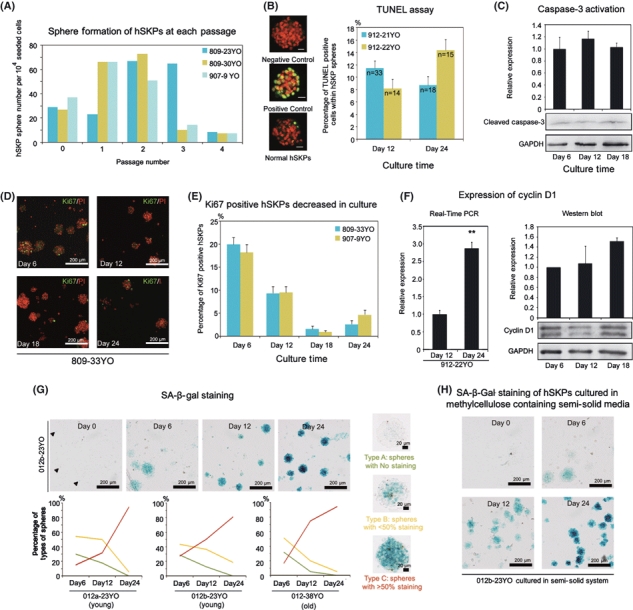
Human foreskin-derived skin-derived precursors (hSKPs) quickly senesced in culture and lost their self-renewal ability. (A) Sphere-forming efficiency of hSKPs from three independent donors (23, 30, and 9 years old) at different passages. (B) TUNEL assay of hSKPs on Day 12 and Day 24 of culture. On the left shows representative staining of hSKPs incubated without Enzyme Solution as a negative control (the upper panel), DNase-treated hSKPs as a positive control (the middle panel), and normal hSKPs with apoptotic cells labeled green (the lower panel). PI was used for counterstaining. The histogram on the right shows TUNEL-positive hSKP percentages of two independent donors (21 and 22 years old) on Day 12 and Day 24. Scar bar = 20 μm. (C) Western blot assay of cleaved caspase-3 levels in hSKPs on Day 6, Day 12, and Day 18, which showed no statistically significant difference. The upper panel shows the statistical results of gray-scale analysis of three independent experiments, and lower panels show a representative Western blot result. (D) Immunostaining of Ki67 of hSKPs on Day 6, Day 12, Day 18, and Day 24 from a representative culture (donor age = 33). PI was used for counterstaining. (E) Statistical results of Ki67-positive hSKP percentages on Day 6, Day 12, Day 18, and Day 24 of cultures from two donors (33 and 9 years old). (F) Expression of cyclin D1 in hSKPs at different culture time. The left panel shows the relative expression of cyclin D1 mRNAs on Day 12 and Day 24 in hSKPs from a 22-year-old donor (by quantitative real-time PCR assay). Double asterisks mean *P* < 0.01 when Day 24 was compared with Day 12. The histogram on the right shows the statistical results of relative expression of cyclin D1 protein on Day 6, Day 12, and Day 18 in hSKPs from three independent cultures by Western blot, with the result from a representative culture attached below. (G) SA-β-Gal staining of hSKPs at different culture time. Upper panels show the representative staining of hSKPs from a 23-year-old donor on Day 0, Day 6, Day 12, and Day 24. Right panels show typical staining patterns of Type A, Type B, and Type C spheres. The three line charts at the bottom show the percentages of each type of sphere on Day 6, Day 12, and Day 24 from three independent donors. Two donors were young adults (both 23 years old) and the other was older (38 years old). Green lines stand for Type A spheres. Yellow lines stand for Type B spheres, and red lines stand for Type C spheres. (H) SA-β-Gal staining of hSKPs from a 23-year-old donor cultured in methylcellulose-containing semisolid media on Day 0, Day 6, Day 12, and Day 24. SA-β-gal: senescence-associated beta gal. TUNEL, terminal deoxynucleotidyl transferase dUTP nick end labeling; PI, propidium iodide.

Unlike newborn murine SKPs reported by Toma *et al.* (2001), our adult foreskin-derived SKPs could not sustain long-term self-renewal *in vitro* in suspension. Figure 1A showed the sphere-forming ability of hSKPs from three independent donors, which were 23, 30, and 9 years old at different passages. Sphere-forming efficiency was relatively low at Passage 0 (P0) as a result of cellular heterogeneity in culture and peaked at Passage 1–3, with differences between individual donors. However, sphere-forming rate sharply dropped after P3 or P4 (Fig. 1A). As a result, cell expansion after P4 was extremely poor. Table S1 (Supporting information) shows the detailed information of these three independent cultures. Thus, we carried out a series of assays to elucidate the underlying mechanisms.

Firstly, we checked cell apoptosis. We detected apoptotic hSKPs using TUNEL assay (terminal deoxynucleotidyl transferase dUTP nick end labeling) (Fig. 1B). We found that TUNEL-positive hSKPs only accounted for < 15% of all hSKPs within a sphere. We compared the percentage of apoptotic hSKPs in spheres on Day 12 (P1) and Day 24 (P3). Changes in the apoptotic hSKP ratio were more likely due to variations in different donors rather than culture time or passage. To confirm this, we examined cleaved caspase-3, the active form of caspase-3 as another apoptotic indicator. Western blot results from three samples showed that the level of cleaved caspase-3 did not change during culture, indicating no increase in hSKP apoptosis (Fig. 1C), consistent with TUNEL analysis (Fig. 1B). These data showed that apoptosis did not contribute to the loss of hSKP self-renewal *in vitro*.

Next, we examined cell proliferation of hSKPs using ki67 staining. We observed a general decline in ki67-positive cell ratios over time (Fig. 1D,E), indicating an overall decrease in hSKP proliferation. We examined the expression of a G_1_ phase cyclin, cyclin D1, using quantitative real-time PCR and Western blot. Cyclin D1 expression was elevated during culture (Fig. 1F), which together with decreased proliferation suggested G_1_ phase cell cycle arrest. This implied the phenomenon of cellular senescence, which is characterized by G_1_ phase arrest (Garkavtsev *et al.*, 1998) and cyclin D1 up-regulation (Han *et al.*, 1999).

Therefore, we investigated cellular senescence of cultured hSKPs. Senescence-associated beta gal (SA-β-gal) expression was evident in cultured hSKPs, indicating hSKPs were undergoing senescence. To study the time-dependent senescence in hSKPs, we compared SA-β-gal staining in freshly isolated dermal cells containing putative hSKPs (Day 0), Day 6 hSKPs, Day 12 hSKPs, and Day 24 hSKPs. We observed that although a minority of Day 0 cells showed weak staining (indicated by arrow heads, upper panel, Fig. 1G), most of the cells were unstained. Day 6 spheres showed evident staining and staining became stronger later on (upper panels, Fig. 1G). To quantitate this change, we categorized hSKP spheres into three types according to the extent of SA-β-gal staining. Type A were spheres with nearly no staining; Type B were spheres with moderate staining (< 50% cells showed staining within a sphere); Type C were spheres with intense staining (≥ 50% cells showed staining within a sphere) (right panels, Fig. 1G). We compared portions of each type of sphere at different time points and made a time-dependent curve. As shown in the lower panels, Fig. 1G, in hSKPs derived from three independent donors, Type A and Type B spheres decreased over time (green and yellow lines) while Type C spheres increased sharply (red lines). For hSKPs from the older donor (38 years old), the change during first 12 days was greater than those from the other two donors (both 23 years old), as indicated by much smaller Type A portion and bigger Type C portion on Day 12. These results raised the possibility that hSKPs from older donors might have a more rapid senescence process *in vitro*.

It was reported that methylcellulose-containing semisolid media could promote cellular expansion and provided a better culture system for hSKPs (Gago *et al.*, 2009). We also assessed cellular senescence in semisolid system. As shown in Fig. 1H, hSKPs still senesced in the semisolid system, indicated by increasing SA-β-gal staining over time. We compared SA-β-gal staining of hSKPs in liquid culture and semisolid culture. Human foreskin-derived SKPs from two out of three donors that we studied showed reduced senescence (bigger Type A portion and smaller Type C portion) in the first 12 days, but Type C spheres became predominant on Day 24 in hSKPs from all the three donors, in both liquid and semisolid cultures (Fig. S2). This result indicated that methylcellulose culture could not prevent hSKP senescence. We also assessed cellular senescence when hSKPs were cultured at relatively low densities. As shown in Fig. S3, hSKPs passaged at 10^4^ cells cm^−2^ also showed considerable SA-β-gal staining at Day 12, indicating that lower densities could not prevent hSKP senescence, either. These data suggested that hSKP senescence *in vitro* could not be prevented simply by the change of culture method. However, SKPs derived from neonatal murine skin showed much weaker SA-β-gal staining at Day 12 of culture (Fig. S4), indicating they were more resistant to *in-vitro* senescence compared with adult human SKPs.

**Fig. 2:**
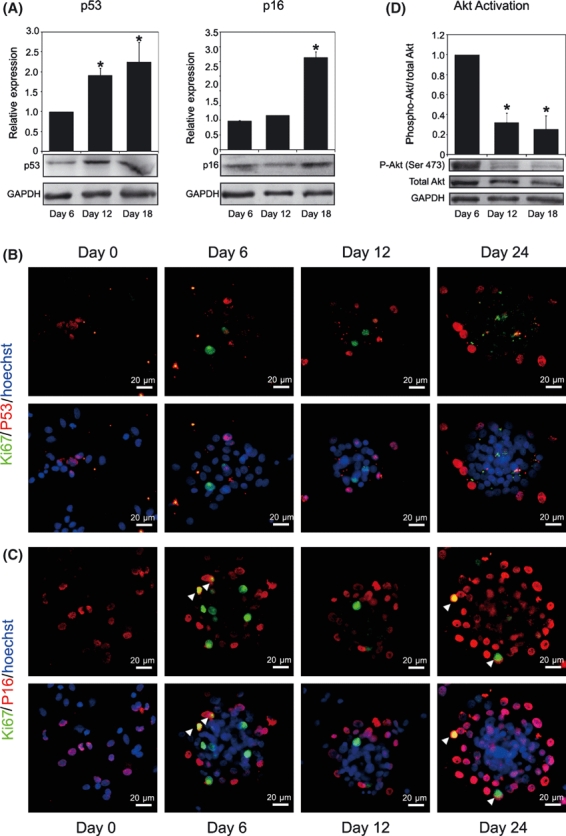
Molecular events in human foreskin-derived SKP (hSKP) senescence. (A) Western blot assay showing relative expression of p53 (left) and p16 (right) in hSKPs on Day 6, Day 12, and Day 18. Histograms show statistical results of gray-scale analysis of three independent cultures, and representative immunoblots are attached below. Asterisk means *P* < 0.05 when compared with Day 6. (B) Dermal cells on Day 0 and hSKP spheres on Day 6, Day 12, and Day 24 immunostained for Ki67 (green) and p53 (red) and counterstained with Hoechst (blue). Upper panels only show channels for Ki67 and p53. Lower panels show channels for Ki67, p53 and Hoechst labeled nuclei. (C) Dermal cells on Day 0 and hSKP spheres on Day 6, Day 12, and Day 24 immunostained for Ki67 (green) and p16 (red) and counterstained with Hoechst (blue). Upper panels only show channels for Ki67 and p16. Lower panels show channels for Ki67, p16, and Hoechst labeled nuclei. White arrowheads indicates cells positive for both Ki67 and p16. (D) Western blot results showing Akt phosphorylation in hSKPs on Day 6, Day 12, and Day 18. The histogram shows the statistical results of gray-scale analysis of three independent cultures and representative immunoblots are attached below. Asterisk means *P* < 0.05 when compared with Day 6.

We further looked into the molecular events underlying hSKP senescence process. P53 and p16 are key regulators of cell cycle and cellular senescence (Beausejour *et al.*, 2003). Western blot results showed that expression of both proteins were up-regulated during culture, supporting the increased senescence of hSKPs (Fig. 2A). Besides, we investigated their time-dependent expression together with the proliferation marker, ki67, at the cellular level to explore their roles in hSKP senescence. Upon isolation, dermal cells containing the putative hSKPs were all negative for Ki67 (green), indicating they were quiescent *in vivo* (Day 0, Fig. 2B,C). A small portion (from none to < 5%, varied among donors) of Day 0 cells showed weak p53 staining (Day 0, Fig. 2B, red), while a bigger portion of cells (from 10% to 30%, varied among donors) were positive for p16 (Day 0, Fig. 2C, red), suggesting that these cells might be undergoing senescence *in vivo*. Both p53- and p16-positive cell portions seemed to increase in hSKP spheres over time (Day 6 to Day 24, Fig. 2B,C), consistent with Western blot results and previously observed increasing senescence shown in Fig. 1G. While p53 was only expressed in a small portion of hSKPs (< 20%), even on Day 24, p16 expression increased sharply over time and extended to most of the cells on Day 24. Moreover, most of Ki67-positive cells were p53 negative, while a subset of them were p16 positive (indicated by co-staining of Ki67 and p16, arrow heads, Fig. 2B,C), indicating that these cycling cells were accumulating p16. We also assessed p21 expression in hSKPs by immunofluorescence, which was also observed in only a small portion of cells (data not shown). These data suggested that hSKP senescence was mainly mediated by intracellular accumulation of p16.

Because of the crucial role of the PI3K-Akt pathway in cell proliferation, survival and senescence, and its regulation on negative regulators of cell cycle, such as p53, p21, and p16, we examined the change of this pathway at different time points. Western blot results showed that Akt phosphorylation decreased sharply on Day 12 and remained low afterward (Fig. 2D), indicating quickly weakened Akt activity in culture in concomitant with the rapid senescent process.

### Blockage of the PI3K-Akt pathway increased hSKP senescence and suppressed hSKP self-renewal

**Fig. 3:**
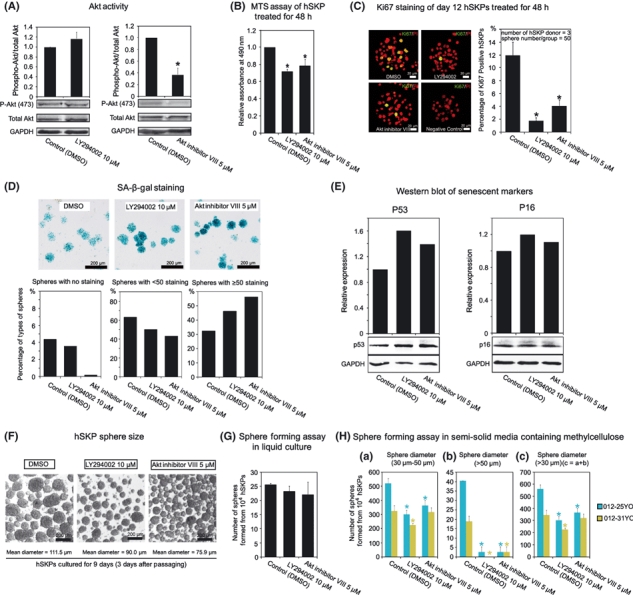
Effects of PI3K and Akt inhibitors on human foreskin-derived skin-derived precursors (hSKPs). (A) Western blot results showing Akt phosphorylation in hSKPs cultured with 0.1% DMSO as a control and 10 μm LY294002 (the left panel) or 5 μm Akt inhibitor VIII (the right panel). Histograms show statistical results of gray-scale analysis of three independent cultures and representative immunoblots are attached below. Asterisk means *P* < 0.05 when compared with control. (B) MTS assays of hSKPs treated with DMSO (as a control), LY294002 or Akt inhibitor VIII for 48 h. The statistical results were derived from three independent experiments. Asterisk means *P* < 0.05 when compared with control. (C) Proliferation of hSKPs after treatment with DMSO (as a control), LY294002 or Akt inhibitor VIII for 48 h shown by Ki67 staining. Left panels show representative hSKP sphere staining and the histogram on the right shows the statistical results from three independent cultures. Asterisk means *P* < 0.05 when compared with control. (D) SA-β-Gal staining of hSKPs cultured with DMSO (as a control), LY294002 or Akt inhibitor VIII for 12 days. Upper panels show representative staining in different groups and lower histograms show the percentages of three types of spheres described previously. (E) Western blot assay showing relative expression of p53 (the left panel) and p16 (the right panel) in hSKPs cultured with DMSO (as a control), LY294002, or Akt inhibitor VIII for 12 days. Histograms show the results of gray-scale analysis and representative immunoblots are attached below. (F) Morphology of hSKP spheres formed in conditions with DMSO (as a control), LY294002 or Akt inhibitor VIII in a typical culture. Group names are labeled on the top and mean diameters of spheres are given at the bottom. (G) Sphere-forming efficiency of hSKPs cultured with DMSO (as a control), LY294002 or Akt inhibitor VIII in liquid culture system. The efficiency was presented as number of spheres formed form 10^4^ cells. (H) Sphere-forming assay of hSKPs treated with inhibitors of the PI3K-Akt pathway in semisolid media containing methylcellulose. (a) Number of spheres with diameters from 30 to 50 μm formed from 10^4^ hSKPs in the presence of 0.1% DMSO (control), 10 μm LY294002 or 5 μm Akt inhibitor VIII. (b) Number of spheres with diameters > 50 μm formed from 10^4^ hSKPs in the presence of 0.1% DMSO (control), 10 μm LY294002, or 5 μm Akt inhibitor VIII. (c) Number of spheres with diameters > 30 μm formed from 10^4^ hSKPs in the presence of 0.1% DMSO (control), 10 μm LY294002 or 5 μm Akt inhibitor VIII. Sphere number of each treatment in c equals the combination of those in a and b. Data show the results derived from a 25-year-old donor (blue columns) and a 31-year-old donor (yellow columns). Each treatment had triplicates, and results were statistically analyzed. Asterisk means *P* < 0.05 when compared with control. MTS, (3-(4,5-dimethylthiazol-2-yl)-5-(3-carboxymethoxyphenyl)-2-(4-sulfophenyl)-2H-tetrazolium).

Concomitant senescence of hSKPs and decrease in Akt phosphorylation suggested a possible role of the PI3K-Akt pathway in the maintenance of hSKPs *in vitro*. Therefore, we tried to block this pathway using a PI3K inhibitor LY294002. However, at the end of the 12 days of culture, we did not detect the inhibition of Akt phosphorylation in hSKPs cultured with 10 μm LY294002 (the left panel, Fig. 3A). We assumed that it was possibly because the basal level of Akt phosphorylation on Day 12 was already very low, rendering the down-regulation difficult to detect. LY294002 could in fact inhibit the growth factor-induced Akt phosphorylation in hSKPs (Fig. S5). Also, we used an inhibitor of Akt1/2, ‘Akt inhibitor VIII’ to confirm the specificity of the Akt pathway. As shown in the right panel, Fig. 3A, a considerable inhibitory effect of Akt inhibitor VIII (5 μm) on Akt activity in hSKPs after 12 days was detected.

After a series of assays, which will be discussed in detail below, we found that both inhibitors substantially impaired hSKP self-renewal even though LY294002 might not show a visible inhibition in Akt activity in long-term culture. First of all, MTS (3-(4,5-dimethylthiazol-2-yl)-5-(3-carboxymethoxyphenyl)-2-(4-sulfophenyl)-2H-tetrazolium) assay was used to study hSKP growth as it is a commonly used homogeneous, colorimetric method for determining the viability of cells and often used as a cell proliferation surrogate. MTS assay showed significant decrease in hSKP growth after LY294002 and Akt inhibitor VIII treatment for 48 h (Fig. 3B). Ki67 staining further confirmed that cell proliferation was suppressed by the two inhibitors after the 48-h treatment (Fig. 3C).

Next, to answer whether blocking the PI3K-Akt pathway would aggravate cellular senescence of hSKPs, we performed SA-β-Gal staining on hSKPs cultured with or without LY294002 and Akt inhibitor VIII for 12 days. LY294002 (10 μm) and Akt inhibitor VIII (5 μm) decreased the portions of spheres with no staining and moderate staining (Type A and B) while increasing the portion of spheres with intense staining (Type C) (Fig. 3D), which suggested they could accelerate hSKP senescence. Also, we checked the expression of senescence-related markers p53 and p16 by Western blot assay. P53 expression (the left panel, Fig. 3E) was up-regulated after inhibitor treatment while p16 expression (the right panel, Fig. 3E) was not obviously altered, which was possibly because p16 level was already high without inhibitor treatment.

In routine massive culture, we observed obvious decrease in sphere diameter of hSKPs cultured with LY294002 or Akt inhibitor VIII compared with control (90.0 and 75.9 vs. 111.5 μm) (Fig. 3F), suggesting impaired sphere formation. Thus, we performed sphere-forming assays to confirm that. However, in liquid culture system, the decrease in sphere-forming efficiency in LY294002 and Akt inhibitor VIII groups was not significant compared with the control group (Fig. 3G). Considering that cell aggregation might confuse true sphere formation in liquid culture, we further assessed sphere formation in methylcellulose-containing semisolid culture to rule out this confusion. Spheres formed 6 days after plating were much smaller than those in liquid culture system. Only spheres with diameters longer than 30 μm were counted. As shown in Fig. 3H, PI3K and Akt inhibitors reduced the total number of spheres formed from 10^4^ cells (Fig. 3H-c), and spheres with diameters longer than 50 μm were nearly abolished under such conditions (Fig. 3H-b). Thus, LY294002 and Akt inhibitor VIII could bona fide inhibit hSKP sphere formation at the used doses. Taken together, these results showed that block of the PI3K-Akt pathway suppressed hSKP proliferation and sphere formation while increasing hSKP senescence *in vitro*, which reflected a potential crucial role of this pathway in the maintenance of hSKP self-renewal.

### Treatment with PDGF-AA and bpV(pic) improved self-renewal of hSKPs and alleviated their senescence *in vitro*

**Fig. 4:**
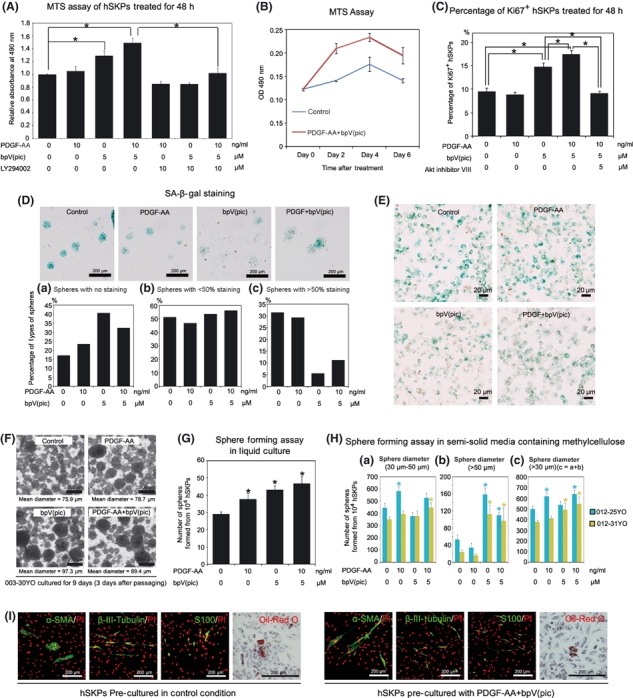
Effects of PDGF-AA and bpV(pic) on human foreskin-derived skin-derived precursors (hSKPs). (A) MTS assay of hSKPs treated for 48 h with PDGF-AA, bpV(pic) or LY294002, in different combinations. Buffer-treated group was used as control. Asterisk means *P* < 0.05 when two labeled groups were compared. (B) MTS growth curves of hSKPs treated with or without the combination of PDGF-AA and bpV(pic) for 6 days. The red line stands for PDGF-AA + bpV(pic) group, and the blue line stands for the buffer-treated control group. (C) Ki67-positive cell percentages in hSKPs treated for 48 h with PDGF-AA, bpV(pic) and Akt inhibitor VIII, in different combinations. Asterisk means *P* < 0.05 when two labeled groups were compared. (D) SA-β-Gal staining of hSKP spheres treated for 12 days without (control) or with PDGF-AA, bpV(pic) and their combination. Upper panels show representative staining in different groups and lower histograms show the percentages of three types of spheres described previously. (E) Representative SA-β-Gal staining of trypsinized hSKPs treated 12 days without (control) or PDGF-AA, bpV(pic) and their combination. Intensity of staining is noteworthy. (F) Morphology of hSKP spheres (from a 30-year-old donor) formed in conditions without (control) or with PDGF-AA, bpV(pic) and their combination. Group names are labeled on the top and mean diameters of spheres are given at the bottom. (G) Sphere-forming assay of hSKPs treated without (control) or with PDGF-AA, bpV(pic) and their combination in liquid culture system. Asterisk means *P* < 0.05 when compared with control. (H) Sphere-forming assay of hSKPs treated with PDGF-AA and bpV(pic) in semisolid media containing methylcellulose. (a) Number of spheres with diameters from 30 to 50 μm formed from 10^4^ hSKPs in the presence of 10 ng mL^−1^ PDGF-AA, 5 μm bpV(pic) or their combination. Buffer-treated group was used as control. (b) Number of spheres with diameters > 50 μm formed from 10^4^ hSKPs in the presence of 10 ng mL^−1^ PDGF-AA, 5 μm bpV(pic) or their combination. Buffer-treated group was used as control. c. Number of spheres with diameters > 30 μm formed from 10^4^ hSKPs in the presence of 10 ng mL^−1^ PDGF-AA, 5 μm bpV(pic) or their combination. Buffer-treated group was used as control. Sphere number of each treatment in c equals the combination of those in a and b. Data show the results derived from a 25-year-old donor (blue columns) and a 31-year-old donor (yellow columns). Each treatment had triplicates, and results were statistically analyzed. Asterisk means *P* < 0.05 when compared with control. (I) Staining of differentiation markers, α-SMA (green), β-III-tubulin (green), S100 protein (green) and Oil-Red O (red), in hSKPs precultured without (control, left panels) or with the combination of PDGF-AA and bpV(pic) (right panels) after a 21-day differentiation. PI or hematoxylin was used for counterstaining. α-SMA, smooth muscle alpha actin; PI, propidium iodide. MTS, (3-(4,5-dimethylthiazol-2-yl)-5-(3-carboxymethoxyphenyl)-2-(4-sulfophenyl)-2H-tetrazolium).

Further, we investigated whether enhancing the PI3K-Akt pathway would improve the growth of hSKPs *in vitro*. We tested several growth factors, chemical compounds, and peptides. According to their receptor expression and effects, we chose PDGF-AA whose receptors were expressed in hSKPs (Fig. S6) and a PTEN inhibitor, bpV(pic). MTS assay of hSKPs cultured with bpV(pic) at different doses for 48 h indicated that 5 and 10 μm bpV(pic) could improve hSKP growth while 30 μm showed cellular toxicity and harmed cell growth (Fig. S7A). Thus, we chose 5 μm as the proper dose. Next, we checked the dose range of PDGF-AA and the combination of PDGF-AA and bpV(pic). As shown in Fig. S7B, higher dose of PDGF-AA (50 ng mL^−1^) had no significant effect compared with 10ng mL^−1^ after 48 h treatment. However, the combination of PDGF-AA and bpV(pic) showed a better mitogenic effect than their separate effects (Fig. 4A). The effects of PDGF-AA and bpV(pic), alone or in combination, were all blocked by 10 μm LY294002 (Fig. 4A), indicating the mediation by PI3K-Akt. As shown in Fig. 4B, during a 6-day culture, growth of hSKPs treated with combination of PDGF-AA and bpV(pic) was improved in comparison with the control. Although the cell number still decreased in both groups on Day 6, this might be caused by absence of medium change during the entire 6-day assay.

Next, cell proliferation was assessed by Ki67 staining. As one can see from Fig. 4C, almost as shown in the MTS assay, bpV(pic) alone had a substantial mitogenic effect while the effect of PDGF-AA alone was not significant as the percentage of Ki67-positive hSKPs was not increased after PDGF-AA treatment. However, PDGF-AA could somehow help improve the effect of bpV(pic), resulting in a better increase in Ki67-positive cell portion in PDGF-AA and bpV(pic) combination group compared with bpV(pic) group. Also, the increase in Ki67-positive hSKP portion by PDGF-AA and bpV(pic) could be counteracted by Akt inhibitor VIII, further confirming the mediation by the PI3K-Akt pathway.

Moreover, we checked the cellular senescence using SA-β-gal staining. hSKP spheres were also divided into three types. As shown in Fig. 4D, bpV(pic) most efficiently increased Type A sphere portion and decreased Type C sphere portion. Besides, the intensity of staining in trypsinized single hSKPs was weakened in bpV(pic) containing groups (Fig. 4E). In contrast, the effect of PDGF-AA alone was not significant, which was in line with other findings above. The SA-β-gal staining results in Figs 1G, 3D and 4D were combined in Fig. S8 for better comparison.

In routine culture, PDGF-AA and bpV(pic) could increase hSKP sphere size (Fig. 4F). Sphere-forming assay in liquid culture showed that both PDGF-AA and bpV(pic) could promote sphere-forming efficiency of hSKPs, and their combined effect was the best (Fig. 4G). Also, sphere formation in semisolid culture indicated that both PDGF-AA and bpV(pic) could increase total sphere number formed from 10^4^ cells (Fig. 4H-c), and bpV(pic), alone or with PDGF-AA, can largely increase the number of spheres with diameters longer than 50 μm (Fig. 4H-b).

To test whether hSKPs remained multipotent after treatment, we differentiated hSKPs cultured for 12 days with or without PDGF-AA and bpV(pic). We employed three differentiation conditions and used α-SMA, β-III-Tubulin, S100 and Oil-Red O to detect smooth muscular, neuronal, glial, and adipocytic cell lineages. After 21-day differentiation, both treated and control hSKPs generated cells positive for these markers (Fig. 4I). Quantitation of marker expression in each condition was shown in Table 1. No statistically significant difference was observed in the derivation of each type of cell from hSKPs precultured with or without PDGF-AA and bpV(pic), implying that treatment of PDGF-AA and bpV(pic) did not change the differentiation potential of hSKPs.

**Table 1:** Quantitation of the percentage of differentiated progeny derived from human foreskin-derived skin-derived precursors cultured in the presence or absence of the combination of PDGF-AA and bpV(pic)

Percentage of differentiated cells positive for a certain marker [control vs. PDGF-AA + bpV(pic)] (Mean ± SEM)			

Differentiation conditions	Condition A	Condition B	Condition C
Marker			
β-III-tubulin	2.9 ± 0.9 vs. 1.8 ± 0.3	7.4 ± 1.3 vs. 12.3 ± 2.4	N/A
α-SMA	12.9 ± 2.8 vs. 18.4 ± 6.7	7.9 ± 1.6 vs. 7.9 ± 1.8	N/A
S100	Rare	13.2 ± 0.4 vs. 14.9 ± 2.7	N/A
Oil-red O	Rare	N/A	35.7 ± 3.8 vs. 35.6 ± 2.1

None of the pairs of data showed statistically significant difference (i.e. *P* > 0.05 in *t*-test).

Condition A: DMEM/F12 with 1% fetal bovine serum (FBS).

Condition B: Neurobasal medium with 1% N2 supplement, 4 μm forskolin, 10 ng mL^−1^ heregulin and 1% FBS.

Condition C: DMEM/F12 with 1 mm dexamethasone, 1 mm isobutylmethylxanthine, 20 μg mL^−1^ insulin and 10% FBS.

N/A, not applicable; α-SMA, smooth muscle alpha actin.

### PDGF-AA and bpV(pic) inhibited key activators of cellular senescence and down-regulated the activity of FoxO3 and GSK-3β

**Fig. 5:**
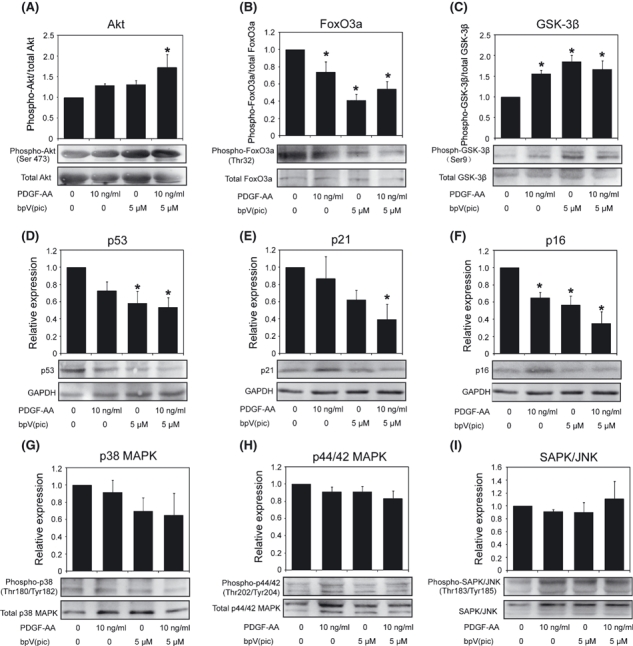
Western blot assay of relevant signal molecules in human foreskin-derived skin-derived precursors (hSKPs) cultured for 12 days in different conditions. (A–C) Phosphorylation of Akt, FoxO3, and GSK-3β in hSKPs cultured without (control) or with PDGF-AA, bpV(pic), and their combination. (D–F) Relative expression of p53, p21, and p16 in hSKPs cultured without (control) or with PDGF-AA, bpV(pic), and their combination. (G–I) Phosphorylation of p38 MAPK, p44/42 MAPK, and SAPK/JNK in hSKPs cultured without (control) or with PDGF-AA, bpV(pic), and their combination. Histograms show statistical results of gray-scale analysis of three independent cultures and representative immunoblots are attached below. Asterisk means *P* < 0.05 when compared with control.

As downstream signal components of Akt such as FoxOs and GSK-3β were reported to be involved in cellular senescence, we investigated changes of these proteins in treated hSKPs. After 12 days of culture, Akt activity was slightly stronger in PDGF-AA and bpV(pic) treated hSKPs than the control group, and the combination of PDGF-AA and bpV(pic) resulted in increased Akt phosphorylation (Fig. 5A). We next analyzed the inhibition of FoxO3 levels, and we found that all treated groups showed lowered FoxO3 phosphorylation indicating reduced activity (Fig. 5B). Likewise, GSK-3β was also inhibited in treated groups, implied by its increased phosphorylation (Fig. 5C). Therefore, both FoxO3 and GSK-3β pathways could be regulated during PDGF-AA- and bpV(pic)-induced hSKP senescence alleviation.

Furthermore, we studied the changes in the expression of several key senescence regulators (Fig. 5D–F). P53, p21, and p16 protein levels were all down-regulated in treated groups. The effect of PDGF-AA alone was not so significant, which was in line with the fact that it only slightly alleviated hSKP senescence. However, it seemed to augment the effect of bpV(pic) as the combination group showed stronger inhibition of these three proteins than bpV(pic) alone.

Given the fact that PDGF-AA could also activate the MAPK pathway, we checked the activity of p38 MAPK, p44/42 MAPK, and SAPK/JNK in treated hSKPs. As shown in Fig. 5G–I, the activity of these MAPKs was not up-regulated in PDGF-AA-treated hSKPs. bpV(pic) treatments seemed to down-regulate p38 MAPK. Based on these results, we propose that the alleviated senescence and promoted self-renewal of hSKP by PDGF-AA and bpV(pic) were not mainly mediated by MAPK pathways.

## Discussion

Although SKPs have received intensive attention from researchers in recent years, only a few studies focused on human SKP regulation and *in-vitro* culture even though some groups did notice that the widely used culture condition could not support a long time expansion of human SKPs (Joannides *et al.*, 2004; Gago *et al.*, 2009). Considering the potential use of hSKPs, a potential dermal stem cell type which could easily be derived from foreskins and other skin biopsies, insights into self-renewal control of human SKPs is important. In this study, we addressed several basic questions.

First, we found that cellular senescence was an important reason for the failure of hSKP expansion *in vitro*. Cellular senescence is a state of growth arrest (Campisi & d'Adda di Fagagna, 2007). Although cellular senescence has been studied intensively, there is a relative lack in the study about stem cell senescence, which is yet important because stem cells maintain many tissues throughout life. Cellular senescence can be triggered by telomere shortening, known as replicative senescence, which happens gradually after many cell divisions (Herbig *et al.*, 2004). In our system, accelerated senescence of hSKPs in culture was not likely induced by cell doubling and telomere shortening. Indeed, cellular senescence can also be triggered by other stimuli, such as DNA damage, oncogene activation, oxidative stress, lack of nutrients or growth factors, and improper cell contacts (Ben-Porath & Weinberg, 2005). It is possible that hSKP senescence might be a physiologically associated phenomenon which is aggravated *in vitro*. Dermal cells already express senescence activating signals (mainly p16) to certain levels *in vivo*, which was in line with the observation by Ressler *et al.* (2006). Augment and accumulation of these signals in hSKPs after separated from their niche might inhibit their mitogenic response to growth factors, and in turn the lead to their senescent phenotype. Our preliminary data also suggested that the *in-vitro* senescence might be related to donor age, but more samples will be required to systematically study this relationship in the future.

Second, we uncovered a role of the PI3K-Akt pathway in hSKPs. As one of the most important signal pathways, the PI3K-Akt pathway has been reported to function in a variety of stem cell types. It was demonstrated that the PI3K-Akt pathway is crucial for the self-renewal of ESCs (Paling *et al.*, 2004), and activation of Akt signaling was sufficient to maintain pluripotency in mouse and primate ESCs (Watanabe *et al.*, 2006). Components of PI3K pathway were also reported to be involved in the self-renewal of neural stem/progenitor cells, with a key role of PTEN as a negative regulator (Groszer *et al.*, 2001, 2006; Gregorian *et al.*, 2009). In the skin, the well-documented role of PI3K-Akt pathway in several types of tumors is thought to be related to stem cells resident in the skin, including epidermal stem cells (Murayama *et al.*, 2007) and skin melanocyte stem cells (Inoue-Narita *et al.*, 2008). Interestingly, SKPs or their derivatives were recently proposed to be the cell origin of dermal neurofibroma (Le *et al.*, 2009). The role of PI3K signaling in neurofibromas (Zhu & Parada, 2002) suggested its potential role in SKP self-renewal. With regard to cellular senescence, different studies revealed different yet important roles of PI3K-Akt signaling as an inhibitor or an activator (Collado *et al.*, 2000; Nogueira *et al.*, 2008). Akt, as a multifunctional signal molecule that governs cell survival, senescence, metabolism, and so forth, could induce different effects in different contexts and eventually act for or against senescence. Here, senescence and loss of self-renewal of hSKPs were accompanied by decreased Akt activity. Further inhibition of Akt phosphorylation aggravated the situation. In contrast, enhanced Akt activation by PDGF-AA and bpV(pic) did reduce hSKP senescence and promoted self-renewal. Future work is needed to elucidate detailed role of the PI3K-Akt pathway in SKP behavior *in vitro* and *in vivo*.

Further, we ameliorated hSKP senescence and improved their self-renewal *in vitro* using a growth factor and a chemical compound, which enhanced the activity of the PI3K-Akt pathway. Although cellular senescence normally acts as tumor-suppressing mechanism in the case of DNA damage and oncogene activation, it could also lead to premature tissue and organ aging (Campisi & d'Adda di Fagagna, 2007), failure of long-term expansion of stem cells *in vitro* (Su *et al.*, 2009) and low efficiency of reprogramming (Esteban *et al.*, 2010). Alleviation of stem cell senescence could be accomplished by the inhibition of senescence activators in aging cells, such as p53 (Karlseder *et al.*, 2002) and p16 (Janzen *et al.*, 2006). Considering future applications, factor supplementation in cell culture is preferred than direct genetic manipulation. Small molecules have been focused on, because of their use in cancer therapy, generation of induced pluripotent cells (iPSCs) and so on. bpV(pic) is a small chemical compound that specifically suppresses PTEN activity (Rosivatz *et al.*, 2006). A recent work showed that it could inhibit PTEN to activate dormant primordial ovarian follicles both in mice and in human (Li *et al.*, 2010). As it is reported that PTEN deletion decreased growth factor dependency of neural stem/progenitor cells *in vitro* (Groszer *et al.*, 2006), it is also worth investigating whether bpV(pic) could decrease the need for growth factors by hSKPs. Interestingly, a recent work promoted the efficiency of iPSC derivation by adding Vitamin C which acted at least in part by inhibiting the activation of p53 and cellular senescence and facilitated reprogramming (Esteban *et al.*, 2010). It is intriguing to investigate in the future whether vitamin C or other factors could also alleviate hSKP senescence and thus optimize culture conditions for hSKPs.

Moreover, we did some preliminary investigation on the signal pathways that regulate hSKP senescence. Signal pathways that govern cellular senescence have been concentrated on, yielding different and seemingly inconsistent results in different systems. Noteworthily, recent studies using gene knockout mice revealed that the loss of some negative regulators of cell cycle, such as p53, actually induced overall aging because of accelerated cell division and premature cell depletion (Tyner *et al.*, 2002). Specifically, TAp63 loss led to premature mSKP senescence and skin aging (Su *et al.*, 2009). Cellular senescence, as a protecting mechanism from and the final consequence triggered by various insults, is governed by a complex regulatory signal network which involves the balance between multiple pathways and feedback mechanisms. We speculate that cells in different status (not senescent, senescing or senescent) have distinct signal patterns and thus respond differently to the modification of senescence regulators. In our system, enhanced activity of Akt in senescing hSKPs could reduce accumulation of p53, p21, and p16 and mitigate hSKP senescence. The effects of Akt on these factors have been well studied. Besides, we found that downstream of Akt, FoxO3, and GSK-3β were inhibited by PDGF-AA and bpV(pic), which might be a direct result of Akt activation. This suggested a potential role of FoxO3 and GSK-3β in senescence inhibition of hSKPs. We also showed that the effect of PDGF-AA and bpV(pic) was not mainly mediated by MAPK pathways.

To summarize, we studied the regulation of hSKPs from an angle of cellular senescence. Our study provided a link among stem cell self-renewal, stem cell senescence and the PI3K-Akt pathway, delivered novel information about hSKP maintenance, and pointed out new directions for future effort concerning the practice of these potential dermal stem cells. Nonetheless, much work still remains to be done to elucidate the detailed mechanisms controlling hSKP self-renewal and senescence in the near future.

## Experimental procedures

### Establishment of hSKP suspension culture *in vitro*

Human foreskin samples were derived from voluntary circumcisions with informed consents, and the protocol was approved by the Ethical Committee of the Institute of Zoology, Chinese Academy of Sciences. Although we obtained some samples from donors older than 50 years of age, most of hSKPs in this study were from donors between 20 and 40 years of age. Foreskin samples were kept in ice-cold sterile saline immediately after surgeries and processed within 5 h. Our method of isolating dermal cells was modified from a previous report (Toma *et al.*, 2005). Briefly, human foreskin biopsies were washed with sterile saline and subcutaneous tissues were cut off followed by additional wash. Samples were cut into pieces of 5 × 5 mm and incubated with 5 mg mL^−1^ Dispase (Gibco, NY, USA, http://www.invitrogen.com) in phosphate-buffered saline (PBS) overnight at 4 °C before the epidermis was ripped off. Dermal pieces were incubated in 5 mg mL^−1^ Collagenase Type IV (Gibco) at 37 °C for 4–8 h until no solid tissue was visible. Single cell suspension was obtained by pipetting and filtering through a 40-μm cell strainer. Cell suspension was centrifuged, washed, plated in growth medium at a density of 10^6^ cells mL^−1^ in 6-cm petri dishes (BD Falcon, CA, USA, http://www.bd.com), and cultured at 37 °C with 5% CO_2_. Basal growth medium was DMEM/F12 (Gibco) supplemented with 20 ng mL^−1^ epidermal growth factor, 40 ng mL^−1^ basic fibroblast growth factor (both from Peprotech, NJ, USA, http://www.peprotech.com), and 2% B27 (Gibco). PDGF-AA (Peprotech), bpV(pic) (Alexis, Switzerland, http://www.enzolifesciences.com/alexis), LY294002 (Sigma, USA, http://www.sigmaaldrich.com), or Akt inhibitor VIII (Sigma) was added in some experiments. Penicillin/streptomycin was used in all culture experiments. After 3 days of culture, cells were transferred to Corning 60-mm ultra-low-attachment culture dishes (Corning, USA, http://www.corning.com/lifesciences) to avoid attachment.

Cell aggregates were seen within 3 days after isolation, and tight spheres were formed on Day 6. For subculture, medium was changed every 3 days and cells were passaged every 6 days. To passage hSKPs, 0.05% trypsin-EDTA (Gibco) was used to disaggregate the spheres. Other cell types were gradually eliminated because of their need for attachment to a surface.

Neonatal murine skin was used to isolate murine SKPs as described by Toma *et al.* (2005). Culture methods for murine SKPs were the same as those for hSKPs.

### Differentiation of hSKPs

After 12 days of culture in growth medium, hSKP spheres were trypsinized and pipetted into single cells. Cells were seeded into 11-mm coverslips precoated with poly-d-lysine/Laminin (Sigma) in 24-well plates. For neuronal differentiation, the medium was Neurobasal medium (Gibco) with 50 ng mL^−1^ nerve growth factor, 50 ng mL^−1^ brain-derived neurotrophic factor, 10 ng mL^−1^ NT-3 (all from Peprotech), and 1–5% fetal bovine serum (FBS) (Hyclone, Australia, http://www.hyclone.com). For Schwann cell condition, the medium was Neurobasal medium with 1% N2 supplement (Gibco), 4 μm forskolin (Sigma), 10 ng mL^−1^ heregulin (Peprotech), and 1% FBS. For adipocyte differentiation, DMEM/F12 containing 10% FBS, 1 mm dexamethasone (Sigma), 1 mm isobutylmethylxanthine (Sigma), and 20 μg mL^−1^ insulin (Sigma) was used. For spontaneous differentiation, the medium was DMEM/F12 with 1–5% FBS. Differentiation took 2–4 weeks depending on morphology of the cells. Medium was half-changed every 3 days.

For quantitative differentiation assays, three different conditions described in Table S1 (Supporting information) were used to assess the derivation of different types of cells. Each condition was duplicated. At the end of a 21-day differentiation, cells were fixed and stained for certain markers. Radom fields were selected and photographed and percentage of cells positive for a certain marker was estimated by dividing the number of cells positive for this marker by the total cell number.

### MTS assay and statistics

Day 12 hSKPs were trypsinized and pipetted into single cells. Cells were seeded into a ultra-low-attachment 24-well plate (Costar, USA, http://www.corning.com/lifesciences) at a density of 10^5^ cells per well in hSKP growth medium plus different factors. MTS assay was performed using CellTiter 96® AQueous One Solution Cell Proliferation Assay (Promega, USA, http://www.promega.com) according to the manufacturer's instructions. MTS assay was applied at 48 h or 0, 24, 48, and 72 h after cell seeding. Each treatment had triplicates and at least three independent experiments were performed for statistics. All data were represented as mean ± SEM. Data were analyzed using Student's *t*-test for comparison between two groups or one-way anova test for more than two groups. A *P*-value of 0.05 was considered significant.

### Semi-solid culture in methylcellulose-containing media

Methylcellulose was purchased from Sigma, and 2% methylcellulose in DMEM/F12 was made as stock solution. A final concentration of 0.8% methylcellulose in DMEM/F12 was used for cell culture.

### Sphere-forming assay and cell counting

To investigate the time-dependent cell growth, cells in each group were seeded at a density of 5 × 10^5^ cells mL^−1^ upon isolation. On Day 6, 12, 18, 24, and 30, sphere numbers were counted and cells were trypsinized into single cells. Cell numbers were counted, and sphere-forming rate at each time point was calculated as the sphere number of 6 days later divided by cell number of the current time point.

For sphere-forming assay, Day 12 spheres were trypsinized into single cells and seeded at a density of 10^4^ cells per well of an ultralow-attachment 24-well plate in different media and cultured for 6 days to investigate the sphere-forming ability of hSKPs in different culture conditions. Each treatment had triplicates and at least three hSKP samples were used for analysis. Statistics were carried out as described above.

Human foreskin-derived SKPs from two independent donors were used for sphere-forming assay in semisolid culture. Media containing 0.8% methylcellulose was used instead of liquid media, and other conditions were the same as in liquid media as described above.

### TUNEL assay

Human foreskin-derived SKP spheres were cytospinned. Cells were fixed and TUNEL Assay was carried out using an *In Situ* Cell Death Detection kit, POD (Roche, USA, http://www.roche-applied-science.com) according to the manufacturer's instructions. For each sphere, total cell number and labeled cell number were counted. Percentage of labeled cells within each sphere was calculated and used for statistics that was performed as described above.

### Immunostaining

For sphere staining, hSKP spheres were cytospinned and fixed with 4% paraformaldehyde (Sigma) in PBS, washed for three times with PBS, and then permeablized and blocked in PBS with 5% BSA (Sigma) and 0.2% triton-X 100 (Sigma) at 37 °C for 1 h. Primary antibodies were applied in PBS with 1% BSA and 0.2% triton-X 100 at 4 °C overnight. After washing the slides with PBS for 3 × 5 min, appropriate secondary antibodies (Zhongshan Goldenbridge Biotechnology, China, http://www.zsbio.com) were applied also in PBS with 1% BSA and 0.2% triton-X 100 at 37 °C for 1 h. Slides were washed 3 × 5 min before propidium iodide (PI) or Hoechst 33342 (both form Sigma) was used for nuclear staining. Differentiated cells on coverslips were fixed and stained as for hSKP spheres. Primary antibodies used were listed in Table S2 (Supporting information).

Ki67-positive cell rate was calculated and data was processed as described for TUNEL assay.

### Oil-red O staining

Differentiated cells on coverslips were fixed with 4% paraformaldehyde in PBS for 2 min, washed with PBS for three times and with 60% isopropanol for two times. Then, cells were incubated in 0.3% Oil-Red O (Sigma) in 60% isopropanol at room temperature for 1 h, followed by being washed for three times with 60% isopropanol and three times in distilled water. Hematoxylin (Zhongshan Goldenbridge Biotechnology) was used for counterstaining in some experiments.

### SA-β-gal staining and result processing

Human foreskin-derived SKP spheres of different passages or treatments were cytospinned. SA-β-gal staining was carried out using a SA-β-gal staining kit (Beyotime Biotechnology, China, http://www.beyotime.com). According to the extent of staining, spheres were divided into three groups: spheres with nearly no staining, spheres with < 50% cells stained blue, and spheres with ≥ 50% cells stained blue. Numbers of spheres in three groups were counted and compared. SA-β-gal staining was also applied for trypsinized hSKPs.

### RNA extraction, reverse transcription, and PCR

Generally, total RNAs were prepared from samples using Trizol reagent (Invitrogen, NY, USA, http://www.invitrogen.com). For small samples, RNeasy Micro Kit (Qiagen, USA, http://www.qiagen.com) was used. cDNAs were synthesized using M-MuLV reverse transcriptase (NEB, China, http://www.neb-china.com).

The procedure of the PCR was as follows: 94 °C for 5 min, 35 cycles of 94 °C for 30 s, gene-specific annealing temperature for 30 s, and 72 °C for 30 s, and 72 °C for 5 min.

Quantitative real-time PCR was carried out using RealMasterMix (SYBR Green) (Tiangen, China, http://www.tiangen.com), and the assay was performed in an ABI Prism® 7000 Sequence Detection System (Appliedbiosystem, USA, http://www.Appliedbiosystem.com). The PCR procedure is as follows: 94 °C for 2 min followed by 40 cycles of 94 °C for 15 s and 60 °C for 1 min.

The primers used were listed in Table S3 (Supporting information).

### Protein extraction and Western blot

Human foreskin-derived SKPs of different passages or treatments were centrifuged and collected. Total protein was extracted using radioimmunoprecipitation (RIPA) lysis buffer completed with phenylmethanesulfonyl fluoride (both from Beyotime Biotechnology). Protein concentration was determined using a BCA Protein Assay kit (Beyotime Biotechnology). About 20-μg protein was loaded for each sample in Western blot. Samples were loaded on 12% sodium dodecyl sulfate polyacrylamide gel and transferred onto polyvinyl difluoride membranes. After blocked with 5% nonfat milk in Tris-buffered saline Tween-20 (TBST pH 7.4) for 1 h at room temperature, membranes were incubated with primary antibodies in blocking solution at 4 °C overnight. After incubating the membranes with appropriate secondary antibodies conjugated to horseradish peroxidase (Zhongshan Goldenbridge Biotechnology), detection was performed with ECL Plus kit Pierce ECL Substrate (Pierce, USA, http://www.piercenet.com). Glyceraldehyde 3-phosphate dehydrogenase (GAPDH) was used as a loading control. Antibodies were listed in Table S4 (Supporting information). Western blot results were subjected to gray-scale analysis using Quantity One™ software (Bio-Rad Laboratories, http://www.bio-rad.com), and at least three independent experiments were used for statistical analysis.

## Supporting Information

**Fig. S1** Isolation and characterization of human foreskin-derived SKPs.

**Fig. S2** Comparison of SA-β-Gal staining of hSKPs cultured in liquid system and semisolid system.

**Fig. S3** SA-β-Gal staining of Day 12 hSKPs with a passaging density of 10^4^ cells cm^−2^.

**Fig. S4** Comparison of SA-β-Gal staining of Day 12 mSKPs and hSKPs.

**Fig. S5** Western blot showing Akt phosphorylation in Day 6 hSKPs treated with different factors.

**Fig. S6** Expression of PDGF receptors in hSKPs.

**Fig. S7** MTS assay of hSKPs treated with different doses of bpV(pic) and PDGF-AA.

**Fig. S8** Combination of SA-β-Gal staining in several figures.

**Table S1** Details of three independent hSKP cultures at the first four passages.

**Table S2** Primary antibodies used in immunostaining.

**Table S3** Primers for PCR and real-time PCR.

**Table S4** Primary antibodies used in Western blot.

As a service to our authors and readers, this journal provides supporting information supplied by the authors. Such materials are peer-reviewed and may be re-organized for online delivery, but are not copy-edited or typeset. Technical support issues arising from supporting information (other than missing files) should be addressed to the authors.
