# Supplementary figures and images for: The PI3K-Akt pathway inhibits senescence and promotes self-renewal of human skin-derived precursors in vitro
Source: Aging Cell. 2011 Aug;10(4):661–74. doi: 10.1111/j.1474-9726.2011.00704.x (PMC3193382; doi:10.1111/j.1474-9726.2011.00704.x)

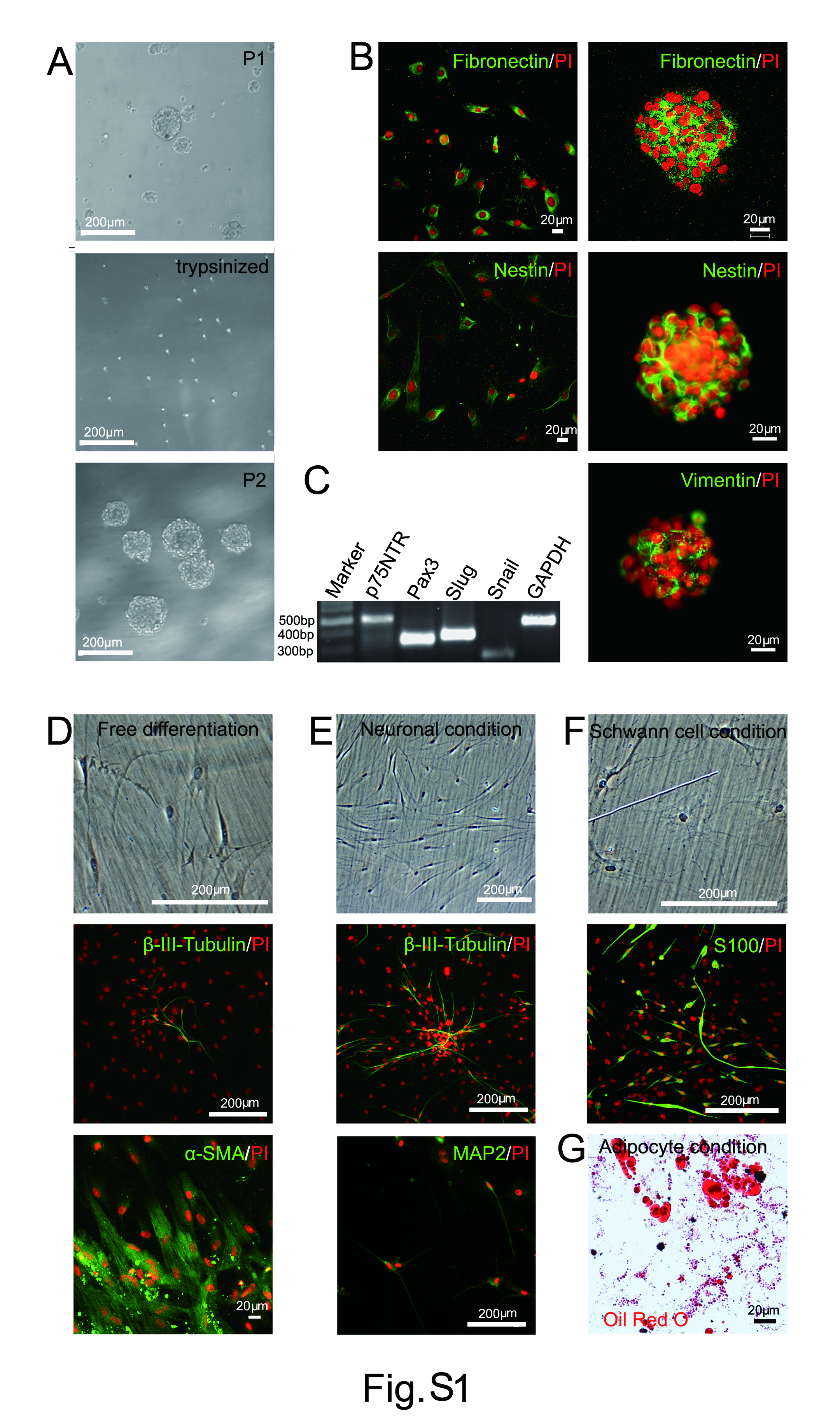

Supplement: Supplementary file 1 [file acel0010-0661-SD1.tif]

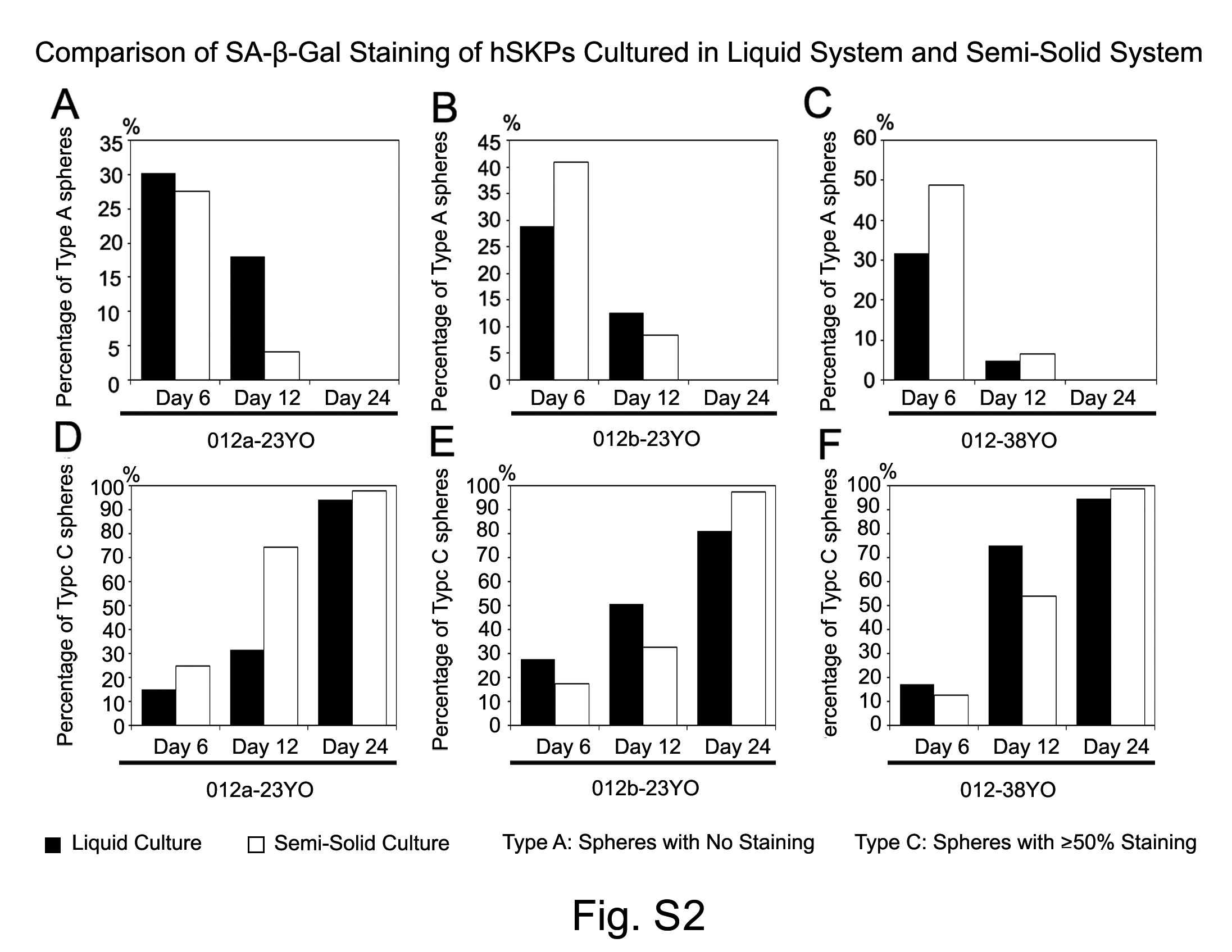

Supplement: Supplementary file 2 [file acel0010-0661-SD2.tif]

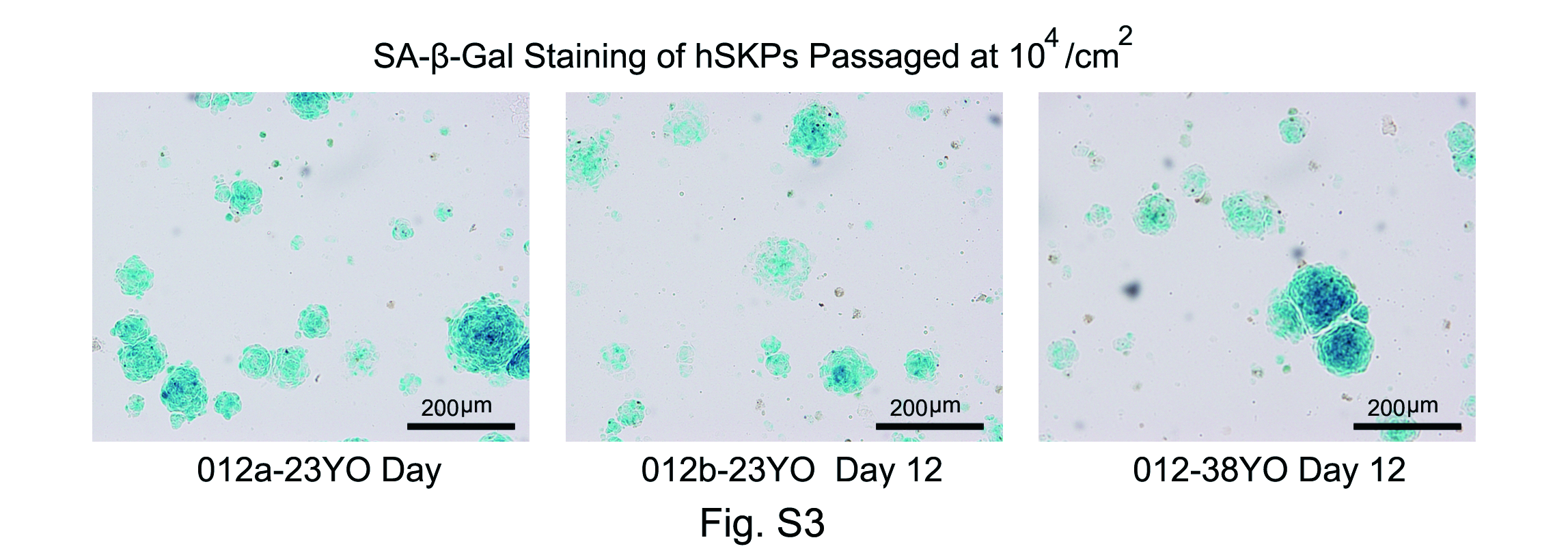

Supplement: Supplementary file 3 [file acel0010-0661-SD3.tif]

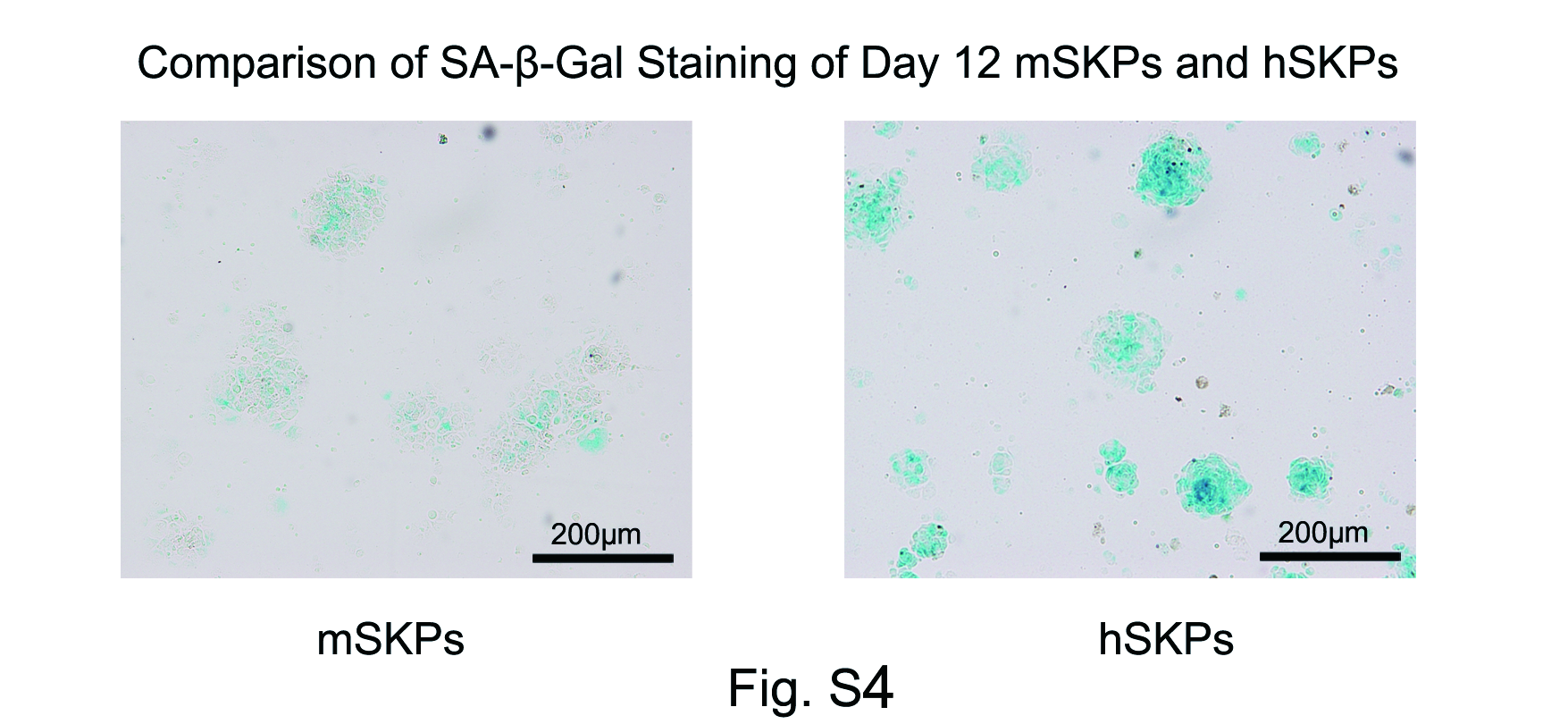

Supplement: Supplementary file 4 [file acel0010-0661-SD4.tif]

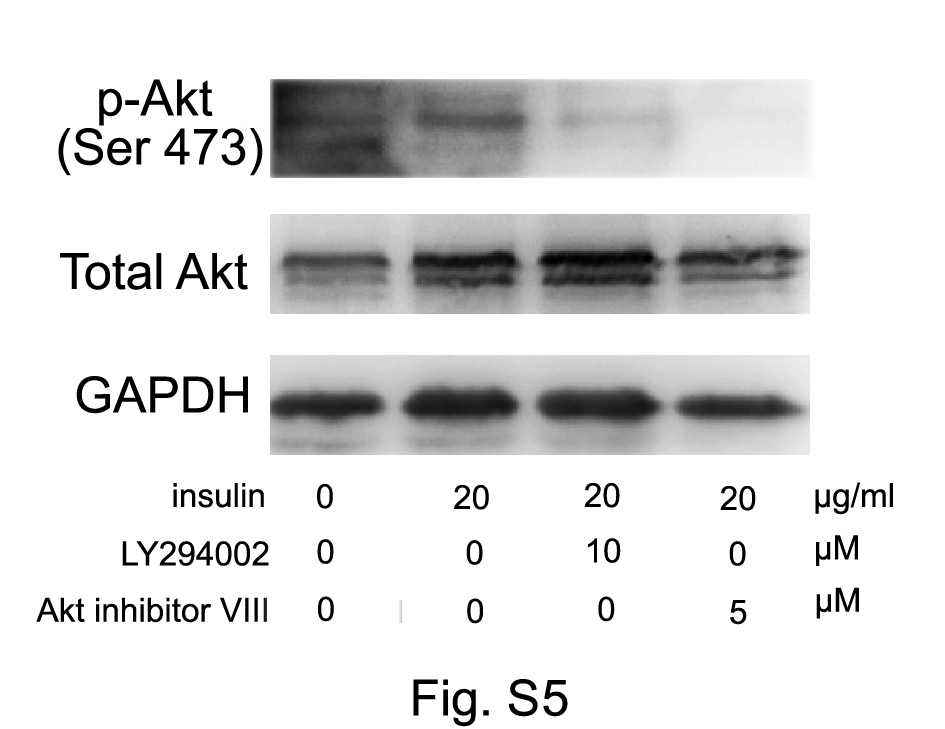

Supplement: Supplementary file 5 [file acel0010-0661-SD5.tif]

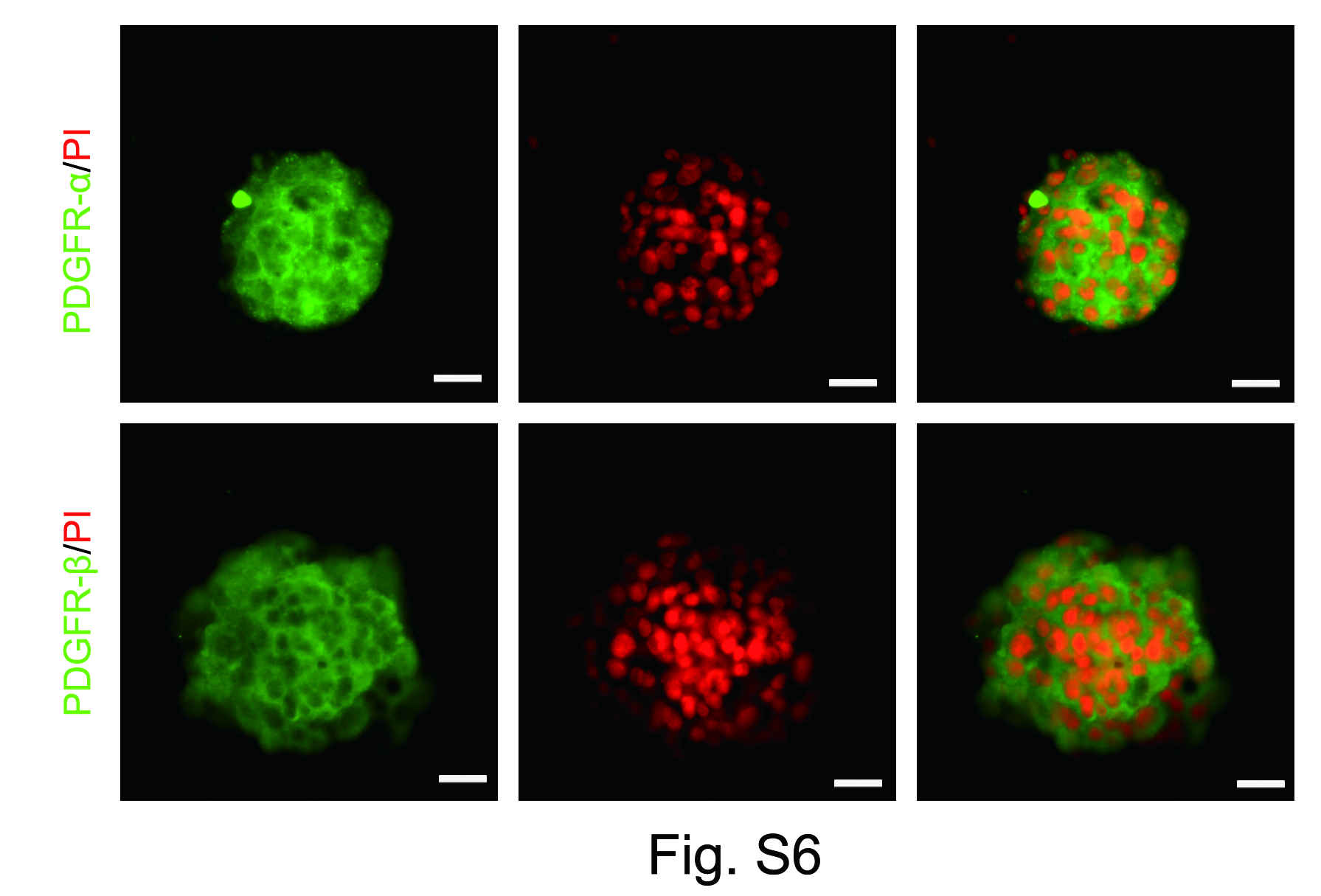

Supplement: Supplementary file 6 [file acel0010-0661-SD6.tif]

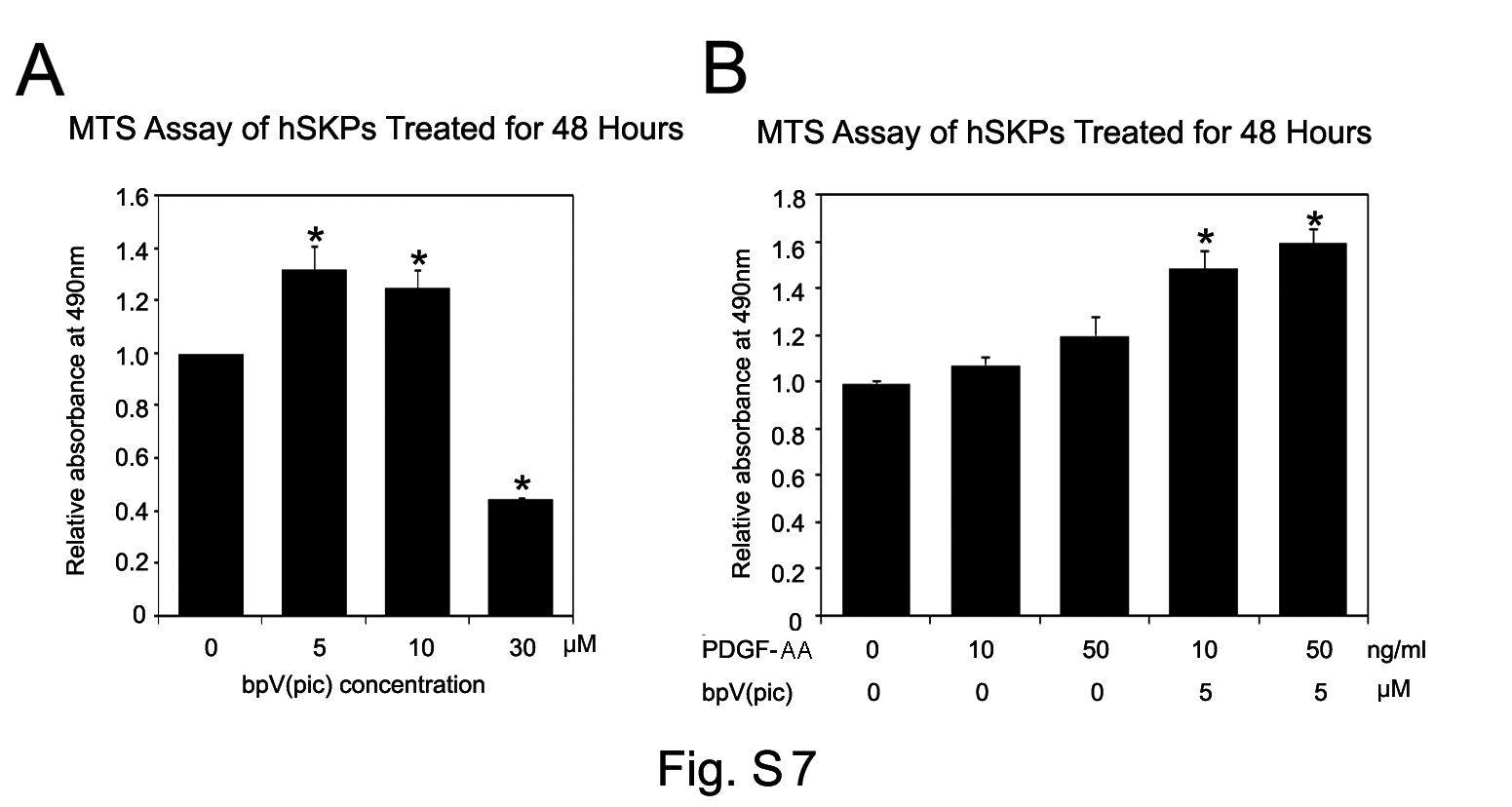

Supplement: Supplementary file 7 [file acel0010-0661-SD7.tif]

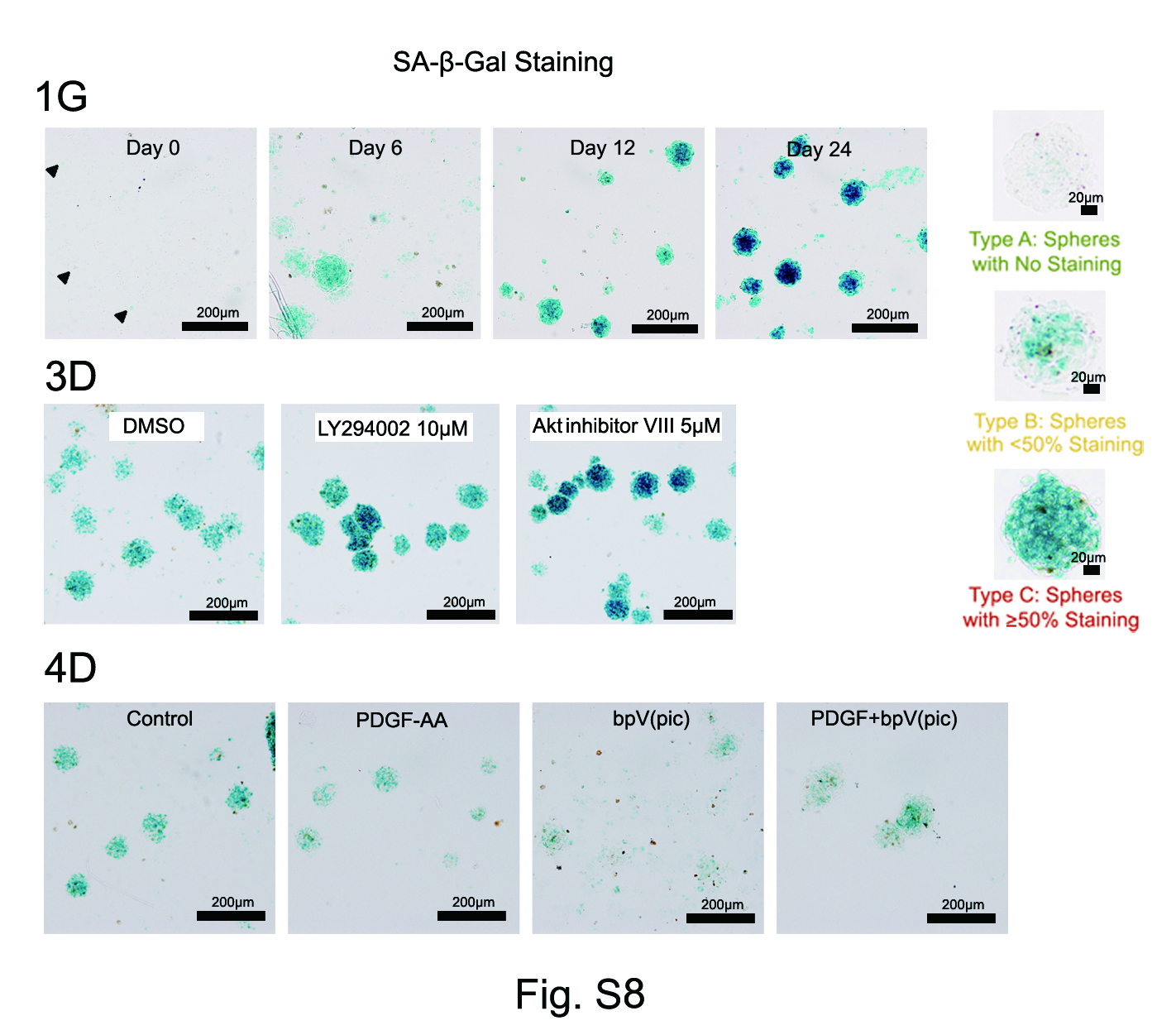

Supplement: Supplementary file 8 [file acel0010-0661-SD8.tif]
