# Supplementary material for: The PI3K-Akt pathway inhibits senescence and promotes self-renewal of human skin-derived precursors in vitro
Source: Aging Cell. 2011 Aug;10(4):661–74. doi: 10.1111/j.1474-9726.2011.00704.x (PMC3193382; doi:10.1111/j.1474-9726.2011.00704.x)
Supplement: Supplementary file 9 [file acel0010-0661-SD9.doc]

| Culture Number | Passage Number | Passage 0 | Passage 1 | Passage 2 | Passage 3 | Passage 4 | Passage 5 |
| --- | --- | --- | --- | --- | --- | --- | --- |
| 809-23YO  Age=23 | Sphere Number | 0 | 6604 | 856 | 817 | 695 | 96 |
|  | Cell Number | 2,250,000 | 366,000 | 122,000 | 107,000 | 110,000 |  |
|  | Sphere Forming Rate = Sphere number at Passage N / cell number at Passage (N-1) * 10,000 | 29.35111 | 23.38798 | 66.96721 | 64.95327 | 8.727273 |  |
|  | **Plating Density (cell number/cm^2^)** | **107,143** | **17,429** | **5,809** | **5,095** | **5,238** |  |
|  | | | | | | | |
| 907-9YO  Age=9 | Sphere Number | 0 | 7428 | 2068 | 760 | 85 | 50 |
|  | Cell Number | 2000000 | 311000 | 148500 | 58125 | 66000 |  |
|  | Sphere Forming Rate = Sphere number at Passage N / cell number at Passage (N-1) * 10,000 | 37.14 | 66.49518 | 51.17845 | 14.62366 | 7.575758 |  |
|  | **Plating Density (cell number/cm^2^)** | **95,238** | **14,810** | **7,071** | **2,768** | **3,143** |  |
|  | | | | | | | |
| 809-30YO  Age=30 | Sphere Number | 0 | 5399 | 970 | 840 | 90 | 43 |
|  | Cell Number | 2000000 | 146000 | 115000 | 86250 | 55000 |  |
|  | Sphere Forming Rate = Sphere number at Passage N / cell number at Passage (N-1) * 10,000 | 26.995 | 66.43836 | 73.04348 | 10.43478 | 7.818182 |  |
|  | **Plating Density (cell number/cm^2^)** | **95,238** | **6,953** | **5,476** | **4,107** | **2,619** |  |

**Table S1. Details of 3 independent hSKP cultures at the first 4 passages.**
