# Supplementary material for: The PI3K-Akt pathway inhibits senescence and promotes self-renewal of human skin-derived precursors in vitro
Source: Aging Cell. 2011 Aug;10(4):661–74. doi: 10.1111/j.1474-9726.2011.00704.x (PMC3193382; doi:10.1111/j.1474-9726.2011.00704.x)
Supplement: Supplementary file 10 [file acel0010-0661-SD10.doc]

**Table S2. Primary antibodies Used in Immunostaining**

| Antibodies | Dilution | Manufacturer |
| --- | --- | --- |
| Fibronectin | 1:200 | Santa Cruz, CA, USA, www.scbt.com |
| Nestin | 1:100 | Santa Cruz, CA, USA, www.scbt.com |
| Vimentin | 1:100 | Santa Cruz, CA, USA, www.scbt.com |
| β-III-Tubulin | 1:100 | Abcam, Cambridge, UK, www.abcam.com |
| α-SMA | 1:100 | Abcam, Cambridge, UK, www.abcam.com |
| MAP2 | 1:50 | Millipore, MA, USA, www.millipore.com |
| GFAP | 1:100 | Millipore, MA, USA, www.millipore.com |
| Ki67 | 1:100 | Neomarkers, CA, USA, www.labvision.com |
| S100 | 1:400 | Santa Cruz, CA, USA, www.scbt.com |
| P53 | 1:200 | Cell Signaling Technology, MA,USA, www.cellsignal.com |
| P16 | 1:100 | Santa Cruz, CA, USA, www.scbt.com |
