# Supplementary material for: The PI3K-Akt pathway inhibits senescence and promotes self-renewal of human skin-derived precursors in vitro
Source: Aging Cell. 2011 Aug;10(4):661–74. doi: 10.1111/j.1474-9726.2011.00704.x (PMC3193382; doi:10.1111/j.1474-9726.2011.00704.x)
Supplement: Supplementary file 11 [file acel0010-0661-SD11.doc]

**Table S3. Primers for PCR and Real-time PCR**

| Gene | Sequence (5’-3’) | Annealing temperature | Product size | GenBank Accession |
| --- | --- | --- | --- | --- |
| p75NTR | TGGGCCCAGAAGGTTGCGATGAA  AAAGGGGCCCCAGAACCAAACACA | 60℃ | 516bp | NM_002507 |
| Pax3 | CATCCGGCCCTGCGTCATCTC  TGGCCTTCTTCTCGCTTTCCTCTG | 60℃ | 352bp | NM_001127366 |
| Slug | CATCTTTGGGGCGAGTGAGTCC  CCCCCGTGTGAGTTCTAATGTGTC | 60℃ | 386bp | NM_003068 |
| Snail | TGGCCTGTCTGCGTGGGTTTTTGT  CCTGGGCTCGGGGCATCTCA | 60℃ | 261bp | NM_005985 |
| GAPDH | GCCAAGGTCATCCATGACAAC  GTCCACCACCCTGTTGCTGTA | 58℃ | 498bp | NM_002046 |
| GAPDH (Real-time PCR) | TGTTGCCATCAATGACCCCTT  CTCCACGACGTACTCAGCG | 60℃ | 202bp | NM_002046 |
| Cyclin D1 | GAACAAACAGATCATCCGCAAAC  GCGGTAGTAGGACAGGAAGTTG | 60℃ | 166bp | NM_053056 |
