# Supplementary material for: The PI3K-Akt pathway inhibits senescence and promotes self-renewal of human skin-derived precursors in vitro
Source: Aging Cell. 2011 Aug;10(4):661–74. doi: 10.1111/j.1474-9726.2011.00704.x (PMC3193382; doi:10.1111/j.1474-9726.2011.00704.x)
Supplement: Supplementary file 12 [file acel0010-0661-SD12.doc]

**Table S4. Primary antibodies used in Western blot**

| Antibodies | Dilution | Manufacturer |
| --- | --- | --- |
| Cyclin D1 | 1:1000 | Santa Cruz, CA, USA, www.scbt.com |
| p53 | 1:1000 | Santa Cruz, CA, USA, www.scbt.com |
| p21 | 1:1000 | Santa Cruz, CA, USA, www.scbt.com |
| p16 | 1:1000 | Santa Cruz, CA, USA, www.scbt.com |
| Akt | 1:1000 | Cell Signaling Technology, MA,USA, www.cellsignal.com |
| Phosphor-Akt (Ser473) | 1:1000 | Cell Signaling Technology, MA,USA, www.cellsignal.com |
| FoxO3 | 1:1000 | Cell Signaling Technology, MA,USA, www.cellsignal.com |
| Phopho-FoxO3 (Thr32) | 1:1000 | Cell Signaling Technology, MA,USA, www.cellsignal.com |
| GSK-3β | 1:1000 | Santa Cruz, CA, USA, www.scbt.com |
| Phospho-GSK-3β | 1:1000 | Cell Signaling Technology, MA,USA, www.cellsignal.com |
| p38 MAPK | 1:1000 | Cell Signaling Technology, MA,USA, www.cellsignal.com |
| Phospho-p38 MAPK (Thr180/Tyr182) | 1:1000 | Cell Signaling Technology, MA,USA, www.cellsignal.com |
| p44/42 MAPK | 1:1000 | Cell Signaling Technology, MA,USA, www.cellsignal.com |
| Phospho-p44/42 MAPK (Thr202/Tyr204) | 1:1000 | Cell Signaling Technology, MA,USA, www.cellsignal.com |
| SAPK/JNK | 1:1000 | Cell Signaling Technology, MA,USA, www.cellsignal.com |
| Phospho-SAPK/JNK (Thr183/Tyr185) | 1:1000 | Cell Signaling Technology, MA,USA, www.cellsignal.com |
| GAPDH | 1:5000 | Millipore, MA, USA, www.millipore.com |
